# Resting state electroencephalography microstates in autism spectrum disorder: A mini-review

**DOI:** 10.3389/fpsyt.2022.988939

**Published:** 2022-12-01

**Authors:** Sushmit Das, Reza Zomorrodi, Peter G. Enticott, Melissa Kirkovski, Daniel M. Blumberger, Tarek K. Rajji, Pushpal Desarkar

**Affiliations:** ^1^Centre for Addiction and Mental Health, Toronto, ON, Canada; ^2^Azrieli Adult Neurodevelopmental Centre, Centre for Addiction and Mental Health, Toronto, ON, Canada; ^3^Temerty Centre for Therapeutic Brain Intervention, Centre for Addiction and Mental Health, Toronto, ON, Canada; ^4^Cognitive Neuroscience Unit, School of Psychology, Deakin University, Geelong, VIC, Australia; ^5^Institute for Health and Sport, Victoria University, Melbourne, VIC, Australia; ^6^Department of Psychiatry, University of Toronto, Toronto, ON, Canada

**Keywords:** electroencephalography, EEG microstates, neurophysiology, autism, mini review

## Abstract

Atypical spatial organization and temporal characteristics, found via resting state electroencephalography (EEG) microstate analysis, have been associated with psychiatric disorders but these temporal and spatial parameters are less known in autism spectrum disorder (ASD). EEG microstates reflect a short time period of stable scalp potential topography. These canonical microstates (i.e., A, B, C, and D) and more are identified by their unique topographic map, mean duration, fraction of time covered, frequency of occurrence and global explained variance percentage; a measure of how well topographical maps represent EEG data. We reviewed the current literature for resting state microstate analysis in ASD and identified eight publications. This current review indicates there is significant alterations in microstate parameters in ASD populations as compared to typically developing (TD) populations. Microstate parameters were also found to change in relation to specific cognitive processes. However, as microstate parameters are found to be changed by cognitive states, the differently acquired data (e.g., eyes closed or open) resting state EEG are likely to produce disparate results. We also review the current understanding of EEG sources of microstates and the underlying brain networks.

## Introduction

Autism spectrum disorder (ASD) is a common neurodevelopmental disorder characterized by persisting impairments in social communication and interaction, and presence of repetitive behaviors, activities and interests (1). Electroencephalography (EEG) microstate analysis in ASD is an emerging field with growing interest in the scientific community. Existing evidence consistently indicates that both functional and structural connectivity are atypical in individuals with ASD (2). These networks include the social brain network (3) (amygdala and superior temporal sulcus) (3), default mode network (4) (posterior cingulate cortex, anterior cingulate cortex, medial prefrontal cortex, medial temporal lobes, angular gyrus, and precuneus) (5, 6), and action observation network (7) (lateral occipital cortex and fusiform gyrus) (7). Studying the spatial and temporal characteristics of these atypical brain networks and their relationship with the core behavioral characteristics of ASD will provide more insights into the neurobiological underpinnings of ASD. EEG is a technique that allows the recording of electrophysiological activity by detecting the fluctuating electrical potentials in the brain (8). One key advantage of quantitative EEG over functional neuroimaging scans is its excellent temporal resolution while investigating the function of large-scale brain networks. While many methods of quantitative EEG analysis exist, microstate analysis provides an unique opportunity to investigate spatial organization and temporal characteristics of large-scale cortical network activities with excellent temporal resolution (9, 10).

**FIGURE 1:**
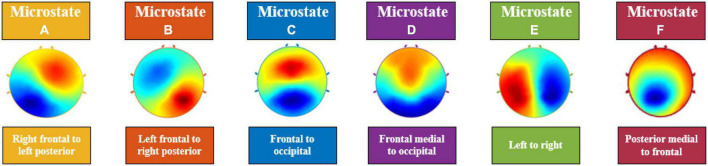
Electroencephalography (EEG) Microstates **(A–F)**. The orientations of the microstates are as follows, **(A)**: right frontal to left posterior; **(B)**: left frontal to right posterior; **(C)**: frontal to occipital; **(D)**: frontal medial to occipital; **(E)**: left to right; **(F)**: posterior medial to frontal.

In a seminal work, Lehman et al. (11) showed that although the topographic maps of resting state EEG signals varied over time, it consisted of few quasi-stable maps called “microstates.” An analysis of 496 participants from ages 6–80 years by Koenig et al. (12) found microstates to be stable for around ∼65–105 ms. Of note, the average microstate duration was found to be higher in children and it decreased with age. It was demonstrated by Liu et al. (13) and Zanesco et al. (14) that depending on the amount of reliable data collected, microstates can show high inter-individual variability. Through cluster analysis, it was noted that segmented EEG signals collapse into temporarily stable spatial patterns; otherwise known as “microstates.” In a recent review, Khanna et al. (15) reports that there are at minimum four main microstate maps consistently reported in the literature, lasting anywhere between 80 and 120 ms before immediately switching to another microstate. These few number of microstates account for 70–80% of all EEG activity during both eyes open and closed resting states and some task related resting state EEG (15). The number of topographical maps are chosen to reflect how well clusters of EEG data resemble them and is denoted by the Global Explained Variance percentage (GEV%) (15). Given the complexity of the central nervous system, having only a handful of these topographical maps explaining up to 80% of resting state EEG data provides a parsimonious account, simplicity, and a compelling reason to study them further. Currently, the four most consistently reported microstates, A, B, C, and D, represent the minimum number of microstates that are revealed by the data (15) (see Figure 1). The orientations of the microstates are as follows, A: right frontal to left posterior; B: left frontal to right posterior; C: frontal to occipital; D: frontal medial to occipital. The lettering conventions are generally accepted in the literature to identify the orientation of the microstates. However, in a recent study, Custo et al. (16) demonstrated that new topographies may be revealed by using a data-driven approach. They also showed that using *a priori* number of clusters could force algorithms to combine microstates, leading to potentially misleading results. Consequently, in recent studies now, microstates E and F are also being reported; with orientations, E: left to right (16); F: posterior medial to frontal (16). Studies can report up to eight microstates in some cases based on their data; however, 4–6 microstates being reported is more common.

### Microstates in psychiatric, neurodevelopmental and neurological disorders

Lehmann et al. (17) originally suggested that these quasi-stable microstates can be interpreted as “atoms of thought,” where different microstates of varying topographies are types or stages of information processing. Subsequently, microstate parameters were linked with personality differences (18) and cognitive ability (19).

Recently, there has been an increasing interest in studying microstates in different psychiatric disorders. It has been found that microstates are significantly altered in diseased states. For example, in Alzheimer’s dementia, the decrease in average duration of each microstate is proportional to cognitive decline (20–22). It was found that microstate A could be the first affected microstate in Alzheimer’s disease (23) and an increase in the duration and frequency of microstate A differentiated patients with Alzheimer’s disease and mild cognitive impairment from healthy participants (23).

A wide variance in topographies and shortening of prominent microstates were found in patients with clinical depression (24). In a recent study, Yan et al. (25) were able to predict the clinical outcomes of major depressive disorder with microstates; a decrease in microstate B duration predicted patient response to antidepressants after 3 months. Further, increased frequency and average duration of microstate A has been found in Tourette’s syndrome and panic disorder, respectively (26, 27). A number of studies also found that an increase in the occurrence of microstates A and C, and a decrease in the occurrence in microstates B and D, correlated with hallucinatory symptoms in patients with schizophrenia (28–30). A meta-analysis by Rieger et al. (31) revealed that the most stable finding in schizophrenia microstate research is an increase in frequency in microstate C and decrease in duration in microstate D.

### Studying electroencephalography microstates in autism spectrum disorder

The study of microstates has its advantages over other forms of EEG analyses. First, EEG signals in general are complex in nature due to their non-stationary and non-linear nature. However, microstate analysis is independent of these two factors, as within a given microstate the dynamics of brain activity are not taken into account. In other words, microstates can consist of any temporal dynamics given that they are consistent throughout their 60–120 ms duration. These microstates can also be studied at any defined frequency band (i.e., delta, theta, alpha, beta, and gamma) (9). As microstates provide a superior temporal resolution in tandem with functional magnetic resonance imaging (fMRI) scan’s high spatial resolution, it provides a unique opportunity to study the brain dynamics in neuropsychiatric disorders with more precision. Three groups, Britz et al. (32), Musso et al. (33), and Yuan et al. (34) have reported different approaches to identify fMRI correlates of EEG resting-state scalp topographies. Recently, Endo et al. (35) reported that fast EEG microstate transitions and slow blood oxygen level-dependent (BOLD) fluctuations changed based on structural connectivity across whole brain region and regions with strong structural connectivity. In another recent a recent study by Abreu et al. (36), EEG microstates were found to reflect large scale brain dynamics collected from fMRI. The group found that the four canonical microstates (i.e., microstates A–D) predicted fMRI dynamical functional connectivity states with 90% accuracy. Lastly, EEG microstates analyses have been shown to have high test–retest reliability. Such analyses provide consistent results with as little as eight electrodes and they have been found to be consistent in different epoch lengths in records with as little as 2 min of recorded EEG data (9, 13).

Khanna et al. (15), suggested that if microstates are thought to reflect coordinated neural activity, then abnormalities can be seen as a breakdown of this activity. There has been consistent evidence that indicates that connectivity in the brain in individuals with ASD is atypical and there is evidence of considerable heterogeneity (37). Further, evidence indicates significant network dysfunction in ASD and some resting state networks (RSNs) such as networks involved with the phonological processing and self-representation, were found to be atypical in ASD (37). Studying spatial and temporal characteristics of these atypical brain networks and their relationship with the core behavioral characteristics of ASD is likely to provide key insights into the neurobiological underpinnings of ASD. Thus, the aim of this mini review is to identify temporal and spatial properties of microstates in ASD and their relationship with cognition and/or core behavioral characteristics in ASD. Further, we will briefly discuss the current evidence of source localizations of EEG microstates and its connection to brain networks and how it relates to ASD.

## Current literature on resting state microstates in autism spectrum disorder

### Method

We searched PubMed, EMBASE and PsychINFO, and included articles published until May 30th, 2022. The following combination of search items was used: EEG, MICROSTATE AND (autism OR autism spectrum disorder OR pervasive developmental disorder OR Asperger disorder OR Asperger syndrome). Additionally, references of selected studies were manually searched. The searched was performed independently by PD and SD and agreement was established after discussion. After screening the title, abstract and full text, articles were included if they studied microstates in ASD. We included studies using human participants and published in English language only.

We recorded the following variables from each article: author, year of publication, diagnosis and comorbidities, study population characteristics (e.g., sample size, sex/gender, age etc.), method used for EEG microstate analysis and, type of EEG recording (eyes open vs. eyes closed rest EEG), number of microstate clusters, microstate parameters (i.e., mean duration, occurrence rate, coverage, and GEV%), relationship of microstate parameters with clinical characteristics, relationship of microstate parameters with cognition and core behavioral characteristics of ASD. This was done by two authors (PD and SD) with disagreements resolved by discussion.

### Findings on resting state microstate parameters in autism spectrum disorder

Microstates extracted from resting state EEGs from the selected studies were found to study brain activity in typically developing (TD) or neurotypical (NT) and ASD groups. The microstates found across the studies explained 72.88–80.44% of the variance of clustering results of ASD and TD or NT EEG data. This percentage represents the clusters at the time samples when global field power (GFP) peaks were calculated; GFP representing the time points with highest signal to noise ratio. Among all microstates, microstate C was found to have the highest GEV% among both groups; however, this was higher in TD groups (25, 38) and NT groups (39).

Across all eight studies, there were significant differences between the TD or NT and ASD groups. In particular, microstate C stood out the most as it was consistently reported to be less frequent in the ASD group in three studies (40–42) (see Tables 1, 2). This finding was consistent even during free viewing of cartoons displaying social interactions (40). Jia and Yu (38) and D’Croz-Baron et al. (39) reported decreased microstate C duration, coverage (39) and GEV% (39) in the ASD group. Nagabhushan Kalburgi et al. (42) also reported increased duration of microstate C in the ASD group. Microstate B also stood out as 4 studies found duration (42, 43), occurrence (38, 39, 43) rate, coverage (39, 43), and GEV% (39, 43) of microstate B to be higher in the ASD group across different age groups (see Tables 1, 2). There were discrepancies regarding the remaining microstates. A higher duration of microstate A was reported in the TD group by Jia and Yu (38). Furthermore, Jia and Yu (38) reported higher microstate D occurrence rate in the ASD group and Nagabhushan Kalburgi et al. (42) reported higher duration in ASD and higher occurrence in TD group. D’Croz-Baron et al. (39) reported higher occurrence, coverage and GEV% of microstate E in the ASD group and Bochet et al. (43) reported higher coverage, occurrence and GEV% of microstate E in the TD group; although it did not survive false discovery rate correction (43).

**TABLE 1:** Summary of key findings related to microstate analysis in ASD research.

Citation	Objective	Sample size	Clinical assessments in ASD group	Microstate and statistical parameters	Results and key findings to microstates
Malaia et al. (62)	To characterize spatiotemporal network dynamics in EEG of ASD and TD groups	14 Diagnosis of Asperger syndrome or high-functioning ASD Individuals (does not specify sex) (age 8–15) 14 TD age and sex-matched participants	**ASD:** Pragmatic Language Observation Scale Score 84 and below **TD:** Pragmatic Language Observation Scale Score 90 and above.	(1) Mean duration of quasi-stable networks (ms) (2) Mean number of quasi-stable networks (3) Mean quasi-stable network diameter	ASD group had a larger mean network diameter and longer mean duration of quasi-stable networks during resting state Higher frequency of quasi-stable networks during resting state in both TD and ASD groups
D’Croz-Baron et al. (39)	Compare resting state microstates between ASD and TD groups.	15 ASD 16 NT Ages 18–30 years old (Does not specify sex)	No assessments reported.	(1) Mean duration (ms) (2) Frequency of occurrence (3) Fraction of time covered (4) GEV%	First step in the 2-step spatial cluster analysis identified between 4 and 7 microstate maps in individuals Group cluster analysis revealed six microstates that best describe the dataset with ∼80% total variance Microstate B (left frontal to right posterior): higher occurrence, (p-pairwise = 0.008; p-corrected = 0.030); higher coverage (p-pairwise = 0.021; p-corrected = 0.063); higher GEV% (p-pairwise = 0.018; p-corrected = 0.054) in the ASD group. Microstate C (frontal to occipital): higher duration (p-pairwise = 0.026; p-corrected = 0.156), coverage (p-pairwise = 0.042; p-corrected = 0.084) and GEV% (p-pairwise = 0.049; p-corrected = 0.098) in the TD group. Microstate E (left to right): higher occurrence (p-pairwise = 0.010; p-corrected = 0.030); coverage (p-pairwise = 0.008; p-corrected = 0.048); GEV% (p-pairwise = 0.010; p-corrected = 0.054) in the ASD group. ASD and TD GEV% (6 maps) = ∼80% ASD and TD GEV% (4 maps) = ∼76%
Jan et al. (40)	Investigate neural activation between ASD and TD groups during free viewing of dynamic semi-naturalistic stimuli containing social interactions.	14 male ASD 14 male TD Ages 2–5 years old	**ASD:** ADOS-2/ADOS-G: 7.4 ± 1.9	(1) Mean duration (ms) (2) Frequency of occurrence (3) Fraction of time covered (4) GEV%	Group-level k-means cluster analysis identified four microstates maps; Significant difference within maps with respect to mean duration, frequency of occurrence, fraction of time covered and GEV% (*p* < 0.001) ASD GEV% = 80.44% TD GEV% = 77.43%
Jia and Yu (38)	Compare resting state microstates between ASD and TD groups.	15 male ASD (mean age = 11.6 years, SD = 4.4) (age 5–18 years) 18 male TD (mean age = 8.9 years, SD 2.4 years) (aged 5–15 years)	**ASD:** Schedule for Affective Disorders and Schizophrenia–Children’s version and (ADOS). All ASD patients were reported to be high-functioning (i.e., IQ > 66). No score reported.	(1) Mean Duration (ms) (2) Frequency of Occurrence (3) Fraction of time covered (4) GEV%	Microstate A: higher duration in TD group; (*p* = 0.006) Microstate B (left frontal to right posterior): higher occurrence, (*p* = 0.001); higher coverage%, (*p* = 0.012) in the ASD group. Microstate C (frontal to occipital): higher duration, (*p* = 0.002); higher coverage%, (*p* = 0.008) in the TD group. Microstate D (frontal medial to occipital): higher occurrence in the ASD group; (*p* = 0.013) ASD GEV% = 78.60% (SD = 2.67%) TD GEV% = 77.11% (SD = 3.08%)
Nagabhushan Kalburgi et al. (42)	To examine eyes-closed and eyes-open resting state EEG microstates of ASD and TD groups	11 male ASD, 2 female ASD (age = 9.7 ± 1.5) 11 male TD, 2 female TD (age = 10.4 ± 1.4)	**ASD:** ADOS-2: 7.8 ± 1.6. 6 of the ASD participants took various ADHD medications; 2 of these 6 also took SSRIs, 1 took risperidone. SCQ: 17.7 ± 6.2 SRS T-score: 73 ± 10 RBS-R: 34.9 ± 21.1 in the Repetitive Behavior Scale–Revised. ASD group had an Abbreviated Battery IQ score of 101.3 ± 19.8 in the Stanford Binet–5 Abbreviated IQ Test. **TD:** SCQ: 3.5 ± 2.5 RBS-R: 1.8 ± 2	(1) Mean duration (ms) (2) Frequency of occurrence (3) Fraction of time covered (4) GEV%	Microstate B (left frontal to right posterior): higher duration (*p* = 0.008) in ASD group Microstate C (frontal to occipital): higher duration (*p* = 0.012) in ASD group in eyes closed condition; higher occurrence (*p* = 0.0014) in TD group in eyes closed condition Microstate D (frontal medial to occipital): higher duration (*p* = 0.004) in ASD group; higher occurrence (*p* = 0.013) in TD group ASD GEV% (eyes closed) = 77.68% (SD = 3.59%) ASD GEV% (eyes open) = 75.08% (SD = 3.26%) TD GEV% (eyes closed) = 73.23% (SD = 4.42%) TD GEV% (eyes open) = 72.88% (SD = 5.00%)
Portnova et al. (63)	To examine difference of resting state EEG between children with low-functioning autism receiving ABA therapy, no ABA therapy and TD group.	10 ASD + ABA therapy group (age = 3.9 ± 1.1) 25 ASD, no ABA (age = 4.1 ± 1.2) 30 TD (age = 4.0 ± 0.9)	**ASD:** ADOS-2: 13.9 ± 3.8 CARS: 43.8 ± 6.8 Non-verbal scale of WPPSI: 101.6 ± 9.9 **ASD + ABA therapy group:** ADOS-2: 14.5 ± 3.7 CARS: 45.2 ± 7.1 Non-verbal scale of WPPSI: 100.7 ± 6.1 **TD:** ADOS-2: 2.7 ± 1.5 CARS: 23 ± 5 Non-verbal scale of WPPSI: 104.6 ± 7.3	(1) Total duration of each cluster (s) calculated before and after ABA therapy	Microstate Cluster left-frontal to right posterior Microstate Cluster right-frontal to left posterior Microstate Cluster parietal
Bochet et al. (43)	Compare resting state microstates between ASD and TD groups.	55 male ASD, 11 female ASD (age = 3.3 ± 1.0) 39 male TD, 8 female TD (age = 3.3 ± 1.2)	**ASD:** ADOS-2/ADOS-G total severity score: 7.67 ± 1.83. MSEL total DQ: 73.4 ± 24.5 **TD:** MSEL total DQ: 110.4 ± 13.7	(1) Mean duration (ms) (2) Frequency of occurrence (3) Fraction of time covered (4) GEV%	Microstate B (left frontal to right posterior): higher GEV%, (*p* < 0.001); higher duration, (*p* < 0.001); higher coverage, (*p* < 0.001); higher occurrence, (*p* < 0.001) in the ASD group. Persisted even when all females were removed from analysis. Survived false discovery rate correction. Microstate E (left to right): higher GEV%, (*p* = 0.031); higher coverage, (*p* = 0.034); higher occurrence, (*p* = 0.019) in the TD group. Did not survive false discovery rate correction. ASD GEV% = 80.8% TD GEV% = 81.9%
Takarae et al. (41)	Compare resting state microstates between ASD and TD groups.	34 male ASD, 4 female ASD (age = 12.19 ± 2.39) 34 male TD, 14 female TD (age = 11.68 ± 3.15)	**ASD:** ADOS total severity score: 11 ± 3.39 VIQ Score: 106.11 ± 18.00 PIQ Score: 105.06 ± 17.96 **TD:** VIQ Score: 109.64 ± 11.75 PIQ Score: 106.15 ± 12.32	(1) Mean duration (ms) (2) Frequency of occurrence (3) Fraction of time covered (4) GEV%	Microstate C (frontal to occipital): higher occurrence (*p* < 0.01) in the TD group. ASD GEV% = 84.05% TD GEV% = 85.28%

ASD, autism spectrum disorder; TD, typically developing; NT, neurotypical; MSEL, mullen scales of early learning; WPPSI, the wechsler preschool and primary scale of intelligence; CARS, child autism rating scale; SCQ, social communication questionnaire; SRS, social responsiveness scale; RBS-R, repetitive behavior scale–revised; IQ, intelligent quotient; PIQ, performance IQ; VIQ, verbal IQ; ABA, applied behavior analysis; GEV%, global explained variance %.

**TABLE 2:** Visualization of key microstate findings.

**Citation**	**A**	**B**	**C**	**D**	**E**	**Microstates**
		
Malaia et al. (62)	Not applicable					Not applicable
D’Croz-Baron et al. (39)		↑ Occurrence, coverage, GEV%	↓ Duration, coverage, GEV%		↑ Occurrence, coverage, GEV%	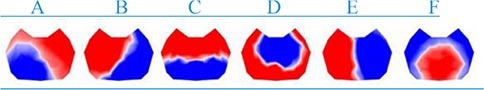
Jan et al. (40)	Significant difference within maps with respect to mean duration, frequency of occurrence, fraction of time covered and GEV%					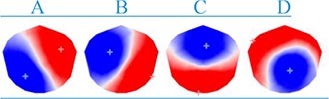
Jia and Yu (38)	↓ Duration	↑ Occurrence, coverage	↓ Duration, coverage	↑ Occurrence		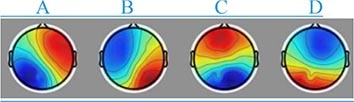
Nagabhushan Kalburgi et al. (42)		↑ Duration	↑ Duration ↓ Occurrence	↑ Duration ↓ Occurrence		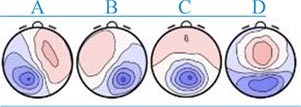
Portnova et al. (63)						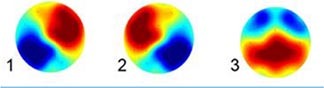
Bochet et al. (43)		↑ Duration, coverage, occurrence			↓ Coverage, occurrence, GEV%	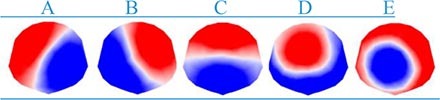
Takarae et al. (41)			↓ Occurrence			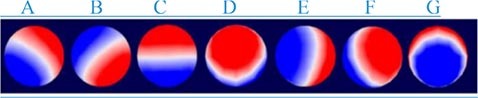

(1) No studies reported any statistical differences between groups with regards to microstates F and G. (2) Eyes closed microstates were extracted from the study by Nagabhushan Kalburgi et al. (3) Microstates extracted represent ASD or ASD + NT/TD only. (4) Arrows represent how ASD data is related to NT/TD.

### Relationship of microstate parameters with clinical characteristics and cognition

Of the eight studies reviewed, two studies were found to include female participants (42, 43). Participants’ ages ranged from 6 months to 30 years old. No relationship between microstate parameters and clinical characteristics such as age, sex, cognition or treatment were reported across the studies studying resting state microstates. No other clinical characteristic was studied in these studies. In the study by Nagabhushan Kalburgi et al. (42), no significant correlation between the mentioned clinical characteristics (see Section “Electroencephalography source localization of microstates”) and microstates was reported. In the study by Bochet et al. (43), the team discovered multiple correlation to clinical measures. First, a negative correlation was found between the Autism Diagnostic Observation Schedule social affects severity score and mean duration of microstate E. Second, a negative correlation was found between the fine motor domain of Mullen Scales of Early Learning and GEV%, coverage, occurrence of microstate D. Next, a positive correlation was found between children’s affective problems in the Child Behavior Checklist and GEV%, coverage, occurrence of microstate B. Lastly, a negative correlation between children’s attention deficits and hyperactivity problems in the Child Behavior Checklist and duration of microstate C. However, it is worth noting that these findings do not survive false discovery rate corrections and are thus exploratory in nature. Bochet et al. (43) also conducted a male-only subgroup analysis and compared to the larger analysis and concluded that their findings were not biased by gender. Bochet et al. (43) further correlated the age of participants with temporal parameters and reported no correlation. Takarae et al. (41) reported the duration of microstate C positively correlated with age in the TD group but not the ASD group. Takarae et al. (41) reported a significant negative correlation between ADOS Social Communication scores and mean duration of microstate C.

## Discussion

From the studies reviewed here, there seems to be significant alterations in microstate parameters in ASD populations as compared to TD or NT populations. Specifically, increased (42) and decreased microstate C duration (38, 39), coverage (39), occurrence (40–42), and GEV% (39) and increased duration (42, 43), occurrence (38, 39, 43) rate, coverage (39, 43), and GEV% (39, 43) of microstate B in the ASD group. Microstate parameters were also found to change in relation to specific cognitive processes; such as eyes open or eyes closed (see Table 1).

Discrepancies mentioned in Section “Future research directions” (i.e., findings related to microstates A, D, and E) could be related to several factors such as age, sample size, states of cognition, and recording length, as more recorded EEG data leads to higher reliability and reproducibility (see Tables 1, 3). For example, Liu et al. (13) found that microstate parameters are revealed with higher intra-class correlation with longer EEG recording length.

**TABLE 3:** Summary of methodology related to microstate analysis in ASD research.

Citation	Microstate analysis method	Sampling rate	Software for microstate analysis	Filtering	Additional filtering approaches (spatial filtering as implemented in Cartool)
Malaia et al. (62)	Network approach to characterization of spatiotemporal dynamics of EEG data in ASD and TD groups	512 Hz	N/a	N/a	N/a
D’Croz-Baron et al. (39)	K-means clustering algorithm (modified version)	500 Hz (Downsampled to 125 Hz)	Cartool Software (64)	1–50 Hz	Cartool spatial filter used
Jan et al. (40)	K-means clustering algorithm	1 kHz (Downsampled to 125 Hz)	Cartool Software (64)	1–40 Hz	Cartool spatial filter used
Jia and Yu (38)	T-AAHC	500 Hz	Cartool Software (64)	2–20 Hz	Cartool spatial filter used
Nagabhushan Kalburgi et al. (42)	T-AAHC	1 kHz	Microstate EEGLab Toolbox (65)	2–20 Hz	N/a
Portnova et al. (63)	K-means clustering algorithm (modified version)	250 Hz	Microstate EEGLab Toolbox (65)	2–30 Hz	N/a
Bochet et al. (43)	K-means clustering algorithm	1 kHz (Downsampled to 125 Hz)	Cartool Software (64)	1–40 Hz	Cartool spatial filter used
Takarae et al. (41)	K-means clustering algorithm (modified version)	256 Hz	Cartool Software (64)	0.5–40 Hz	Cartool spatial filter used

T-AAHC, Topographic Atomize and Agglomerate Hierarchical Clustering.

One major caveat noted is that only three studies (37–39) directly examined the relationship between microstate parameters and clinical characteristics. It is also possible that as no significant difference was found, none was reported in the other studies. D’Croz-Baron et al. (39) commented that a significantly reduced occurrence of microstate C in the ASD group could explain the social impairments faced by the population. In the study by Nagabhushan Kalgurbi et al. (42), no significant correlation between the clinical measures: Autism Diagnostic Observation Schedule-2, Social Communication Questionnaire, Social Responsiveness Scale, Repetitive Behaviors Scale–Revised, Stanford-Binet–5, and microstate parameters were reported. In the study by Bochet et al. (43), multiple correlations were found between clinical measures and microstates B, C, D, and E; however these findings do not survive false discovery rate corrections and thus the authors suggest the findings should be considered exploratory. Taking the findings from Takarae et al. (41) and Bochet et al. (43), it is suggestive that decreased microstate C duration is indicative of greater presence of ASD symptoms.

### Electroencephalography source localization of microstates

In a recent work by Custo et al. (16), using source localization algorithms, the team was able to estimate seven resting-state topography sources. Among these, the four canonical microstates commonly seen were also present. Of note, microstate B saw strong activation in the cuneus and a second weaker activation in the right insula, right claustrum, and right frontal eye field; microstate C saw strong activation in the precuneus, posterior cingulate cortex and a second weaker activation in the left angular gyrus. Interestingly, literature suggests reduced functional connectivity in the precuneus/superior parietal lobule in ASD (44). Furthermore, the posterior cingulate cortex, a region pivotal in cognitive, social and emotional processing, is also reported to be atypical in ASD populations (45). Both hypo- and hyper-connectivity has been reported in insular regions (46), hypo-connectivity between frontal eye fields and dorsal anterior cingulate cortex. Taken together, these preliminary studies reviewed in this paper are suggestive of microstates B and C being crucial to the underlying neurophysiology of ASD.

As mentioned earlier, three groups, Britz et al. (32), Musso et al. (33) and, Yuan et al. (34) have reported different approaches to identify fMRI correlates of EEG resting-state scalp topographies. Although, it is difficult to compare the methods due to different methodological approaches, it still demonstrates that EEG microstates closely describe RSNs identified by fMRI. Of note, Michel and Koenig (10) cautions against making one-to-one attributions of microstates to brain functions revealed by fMRI. Only a handful of other groups (16, 47, 48) in addition to Custo et al. (16) utilized EEG source localization techniques to conclude that the four canonical microstates represent components of the default mode network (48). Pascual-Marqui et al. (48) reports that these four microstates have common posterior cingulate generators, three microstates also had activity in left occipital/parietal, right occipital/parietal and, anterior cingulate cortices. Microstate C as found to be atypical in this review, was reported to have maximum activity in anterior cingulate areas (48); a key atypical area in ASD as discussed previously.

### Future research directions

For future ASD microstate research, one crucial point to take note of is that Custo et al. (16) reports when limiting the number of microstate clusters to 4, both microstates C and F seem to collapse into one. This is important for two reasons. First, it limits new microstates to be discovered when not using a data-driven approach and secondly, because microstate F has strong activations in the dorsal anterior cingulate cortex, superior frontal gyrus, bilateral middle frontal gyrus and bilateral insula. ASD populations been reported to show atypical connectivity in the dorsal anterior cingulate cortex (49), superior frontal gyrus (50), middle frontal gyrus (50), and insula (46) as mentioned above. Since we see consistent atypical parameters in microstate C, it is possible that microstate F once studied may also be atypical in ASD. Coincidently, these regions: posterior cingulate cortex, anterior cingulate cortex, medial prefrontal cortex, angular gyrus and precuneus are implicated in the default mode network. A network that has been extensively studied and reported to be atypical in ASD (51). However, interestingly no groups yet reported any significant findings with regards to microstate F.

Although Bochet et al. (43) reported the findings from the male-only analysis were similar to the larger dataset, the team reported no correlation between microstate parameters and age and sex. Thus, the effect of development and sex on microstate parameters in ASD remains largely unclear. In schizophrenia research, parameters of microstate A and D were found to be significantly altered in younger patient groups (52). Furthermore, in both NT and TD populations, studies have reported significant developmental effect of age and sex over microstate parameters (12, 53, 54). It is worth noting that the ages of participants in Bochet et al. (43) did not vary as much in comparison to all the studies combined (see Table 1). Interestingly, in the study by Takarae et al. (41), the duration of microstate C was reported to positively correlate with 7–19 year old TD and NT participants but not ASD participants. This finding may explain the aberrant parameters of microstate C across studies. The overall variations in microstate findings may still be explained by the large age range, cognition, and core behavioral characters of ASD across the studies. Future studies should also include a large dataset with participants’ ages ranging from infancy to adulthood to clearly investigate the developmental effect of age on microstates in ASD. Future research studies should also be adequately powered and control for some common sources of heterogeneity in autism such as age and sex. There is a need to recruit enough female participants to reflect the 3:1 ratio observed in clinical sample (55). Also, similar to Bochet et al. (43), it would be useful to conduct sex-stratified subgroup analysis to examine the effect and interaction of sex on the microstate parameters. The effect of development on age and sex on microstate parameters should also be studied further in NT and TD populations.

Only three (41–43) identified studies have examined the association between microstates and clinical characteristics, in particular core behavioral characteristics of autism.

There exist opportunities to use machine learning methodologies in resting state microstate analysis. Future studies can further investigate the potential relationship between microstate parameters and clinical characteristics and whether microstates can be used to classify individuals with ASD (12, 54).

There have been numerous studies examining microstates in schizophrenia looking at deviate microstate characteristics, microstate patterns, genomic effects and the association between microstates and symptoms (28, 30, 56, 57). Work is also being done to use microstates as predictors of disease (58). Such methodologies could be used to fill the gap that exists in microstate research related to ASD.

There is potential utility of microstate research to fit into future neuroimaging research, given the increased inter-individual variability and idiosyncrasy seen in the brain in individuals with ASD using neuroimaging data (59). Microstates have also been found to display inter-individual variability. Results reported by Nunes et al. (59) are suggestive of idiosyncratic functional connectivity being a hallmark of the ASD brain. Further, this team also found that the level of idiosyncrasy was associated with core behaviors of ASD. Since both microstates in general and neuroimaging data in ASD display high inter-individual variability, they can be studied in tandem to obtain greater understanding of the heterogeneity and idiosyncrasies in the brain mechanisms in ASD with a higher scale of sensitivity. Such combination will also provide a greater degree of temporal and spatial resolution at the same time. As mentioned previously, recent reports by Endo et al. (35) and Abreu et al. (36) suggest that EEG microstates reflect large scale brain dynamics collected from fMRI and also fluctuate based on structural connectivity along with BOLD fluctuations. Using these approaches, future ASD EEG microstate researchers can study both fast and slow resting-state network dynamics and their associations with different cognitive states and core behavioral characteristics of ASD. One other recent study reported a method to investigate excitation/inhibition regulation in the brain using fMRI, positron emission tomography and EEG microstates (60). One widely cited neurophysiologic model in ASD is altered excitation/inhibition balance in the brain (61); therefore, there is an opportunity to utilize EEG microstate along with fMRI to study the excitation/inhibition balance in the brain in autism with increased precision.

## Conclusion

In summary, the current mini review of microstates in ASD indicates that there is a significant difference between microstates of ASD and TD or NT groups, however the data are heterogeneous, the clinical significance of such altered microstates has not been thoroughly examined, and major sources of heterogeneity in ASD such as age and sex have not satisfactorily been addressed. Combined, these results suggest that EEG microstates can be used to further detect and study atypical functionality in the brain in individuals with ASD. In the future, resting state and event related potential microstate analysis, in tandem with neuroimaging, can be utilized to study atypical functional networks in ASD.

## Author contributions

SD was involved in literature search, reviewing downloaded articles, data extraction, assessment of quality, writing, and reviewing the manuscript. RZ was involved in the design, assessment of quality, writing, and critically reviewing the manuscript. PE and MK were involved in writing and critically reviewing the manuscript. DB and TR were involved in critically reviewing the manuscript. PD was involved in the design, literature search, data extraction, assessment of quality, writing, and critically reviewing the manuscript. All authors approved the submission of this manuscript.

## References

[1] American Psychiatric Association. *Diagnostic and Statistical Manual of Mental Disorders: DSM-5.* 5th ed. Washington, D.C: American Psychiatric Association (2013). 947 p.

[2] Vissers ME Cohen MX Geurts HM. Brain connectivity and high functioning autism: a promising path of research that needs refined models, methodological convergence, and stronger behavioral links. *Neurosci Biobehav Rev.* (2012) 36:604–25. 10.1016/j.neubiorev.2011.09.003 21963441

[3] Sato W Uono S. The atypical social brain network in autism: advances in structural and functional MRI studies. *Curr Opin Neurol.* (2019) 32:617–21. 31135458 10.1097/WCO.0000000000000713

[4] Harikumar A Evans DW Dougherty CC Carpenter KLH Michael AMA. Review of the default mode network in autism spectrum disorders and attention deficit hyperactivity disorder. *Brain Connect.* (2021) 11:253–63. 33403915 10.1089/brain.2020.0865 PMC8112713

[5] Greicius MD Krasnow B Reiss AL Menon V. Functional connectivity in the resting brain: a network analysis of the default mode hypothesis. *Proc Natl Acad Sci.* (2003) 100:253–8. 10.1073/pnas.0135058100 12506194 PMC140943

[6] Supekar K Uddin LQ Prater K Amin H Greicius MD Menon V. Development of functional and structural connectivity within the default mode network in young children. *NeuroImage.* (2010) 52: 290–301. 20385244 10.1016/j.neuroimage.2010.04.009 PMC2976600

[7] Delbruck E Yang M Yassine A Grossman ED. Functional connectivity in ASD: atypical pathways in brain networks supporting action observation and joint attention. *Brain Res.* (2019) 1706:157–65. 10.1016/j.brainres.2018.10.029 30392771

[8] Ingber L Nunez PL. Neocortical dynamics at multiple scales: EEG standing waves, statistical mechanics, and physical analogs. *ArXiv* [Preprint]. (2010). 10.48550/arXiv.1004.4322 21167841

[9] Khanna A Pascual-Leone A Farzan F. Reliability of resting-state microstate features in electroencephalography. *PLoS One.* (2014) 9:e114163. 10.1371/journal.pone.0114163 25479614 PMC4257589

[10] Michel CM Koenig T. EEG microstates as a tool for studying the temporal dynamics of whole-brain neuronal networks: a review. *NeuroImage.* (2018) 180(Pt B):577–93. 10.1016/j.neuroimage.2017.11.062 29196270

[11] Lehmann D Ozaki H Pal I. EEG alpha map series: brain micro-states by space-oriented adaptive segmentation. *Electroencephalogr Clin Neurophysiol.* (1987) 67:271–88. 10.1016/0013-4694(87)90025-3 2441961

[12] Koenig T Prichep L Lehmann D Sosa PV Braeker E Kleinlogel H Millisecond by millisecond, year by year: normative EEG microstates and developmental stages. *NeuroImage.* (2002) 16:41–8. 10.1006/nimg.2002.1070 11969316

[13] Liu J Xu J Zou G He Y Zou Q Gao JH. Reliability and individual specificity of EEG microstate characteristics. *Brain Topogr.* (2020) 33:438–49. 10.1007/s10548-020-00777-2 32468297

[14] Zanesco AP King BG Skwara AC Saron CD. Within and between-person correlates of the temporal dynamics of resting EEG microstates. *NeuroImage.* (2020) 211:116631. 10.1016/j.neuroimage.2020.116631 32062082

[15] Khanna A Pascual-Leone A Michel CM Farzan F. Microstates in resting-state EEG: current status and future directions. *Neurosci Biobehav Rev.* (2015) 49:105–13. 10.1016/j.neubiorev.2014.12.010 25526823 PMC4305485

[16] Custo A Van De Ville D Wells WM Tomescu MI Brunet D Michel CM. Electroencephalographic resting-state networks: source localization of microstates. *Brain Connect.* (2017) 7:671–82. 10.1089/brain.2016.0476 28938855 PMC5736178

[17] Lehmann D Strik WK Henggeler B Koenig T Koukkou M. Brain electric microstates and momentary conscious mind states as building blocks of spontaneous thinking: I. Visual imagery and abstract thoughts. *Int J Psychophysiol.* (1998) 29:1–11. 10.1016/s0167-8760(97)00098-6 9641243

[18] Schlegel F Lehmann D Faber PL Milz P Gianotti LRR. EEG microstates during resting represent personality differences. *Brain Topogr.* (2012) 25:20–6. 21644026 10.1007/s10548-011-0189-7

[19] Santarnecchi E Khanna AR Musaeus CS Benwell CSY Davila P Farzan F EEG microstate correlates of fluid intelligence and response to cognitive training. *Brain Topogr.* (2017) 30:502–20. 10.1007/s10548-017-0565-z 28493012

[20] Dierks T Jelic V Julin P Maurer K Wahlund LO Almkvist O EEG-microstates in mild memory impairment and Alzheimer’s disease: possible association with disturbed information processing. *J Neural Transm Vienna Austria 1996.* (1997) 104:483–95. 10.1007/BF01277666 9295180

[21] Stevens A Kircher T. Cognitive decline unlike normal aging is associated with alterations of EEG temporo-spatial characteristics. *Eur Arch Psychiatry Clin Neurosci.* (1998) 248:259–66. 10.1007/s004060050047 9840373

[22] Strik WK Chiaramonti R Muscas GC Paganini M Mueller TJ Fallgatter AJ Decreased EEG microstate duration and anteriorisation of the brain electrical fields in mild and moderate dementia of the Alzheimer type. *Psychiatry Res.* (1997) 75:183–91. 10.1016/s0925-4927(97)00054-1 9437775

[23] Musaeus CS Nielsen MS Høgh P. Microstates as disease and progression markers in patients with mild cognitive impairment. *Front Neurosci.* (2019) 13:563. 10.3389/fnins.2019.00563 31263397 PMC6584800

[24] Strik WK Dierks T Becker T Lehmann D. Larger topographical variance and decreased duration of brain electric microstates in depression. *J Neural Transm Gen Sect.* (1995) 99:213–22. 10.1007/BF01271480 8579806

[25] Yan D Liu J Liao M Liu B Wu S Li X Prediction of clinical outcomes with EEG microstate in patients with major depressive disorder. *Front Psychiatry.* (2021) 12:695272. 10.3389/fpsyt.2021.695272 34483990 PMC8415359

[26] Kikuchi M Koenig T Munesue T Hanaoka A Strik W Dierks T EEG microstate analysis in drug-naive patients with panic disorder. *PLoS One.* (2011) 6:e22912. 10.1371/journal.pone.0022912 21829554 PMC3146502

[27] Stevens A Günther W Lutzenberger W Bartels M Müller N. Abnormal topography of EEG microstates in Gilles de la Tourette syndrome. *Eur Arch Psychiatry Clin Neurosci.* (1996) 246:310–6. 10.1007/BF02189024 8908413

[28] Lehmann D Faber PL Galderisi S Herrmann WM Kinoshita T Koukkou M EEG microstate duration and syntax in acute, medication-naive, first-episode schizophrenia: a multi-center study. *Psychiatry Res.* (2005) 138:141–56. 10.1016/j.pscychresns.2004.05.007 15766637

[29] Nishida K Morishima Y Yoshimura M Isotani T Irisawa S Jann K EEG microstates associated with salience and frontoparietal networks in frontotemporal dementia, schizophrenia and Alzheimer’s disease. *Clin Neurophysiol.* (2013) 124:1106–14. 10.1016/j.clinph.2013.01.005 23403263

[30] Tomescu MI Rihs TA Becker R Britz J Custo A Grouiller F Deviant dynamics of EEG resting state pattern in 22q11.2 deletion syndrome adolescents: a vulnerability marker of schizophrenia? *Schizophr Res.* (2014) 157:175–81. 10.1016/j.schres.2014.05.036 24962438

[31] Rieger K Diaz Hernandez L Baenninger A Koenig T. 15 years of microstate research in schizophrenia – where are we? A meta-analysis. *Front Psychiatry.* (2016) 7:22. 10.3389/fpsyt.2016.00022 26955358 PMC4767900

[32] Britz J Van De Ville D Michel CM. BOLD correlates of EEG topography reveal rapid resting-state network dynamics. *NeuroImage.* (2010) 52:1162–70. 10.1016/j.neuroimage.2010.02.052 20188188

[33] Musso F Brinkmeyer J Mobascher A Warbrick T Winterer G. Spontaneous brain activity and EEG microstates. A novel EEG/fMRI analysis approach to explore resting-state networks. *NeuroImage.* (2010) 52:1149–61. 10.1016/j.neuroimage.2010.01.093 20139014

[34] Yuan H Zotev V Phillips R Drevets WC Bodurka J. Spatiotemporal dynamics of the brain at rest — exploring EEG microstates as electrophysiological signatures of BOLD resting state networks. *NeuroImage.* (2012) 60:2062–72. 10.1016/j.neuroimage.2012.02.031 22381593

[35] Endo H Hiroe N Yamashita O. Evaluation of resting spatio-temporal dynamics of a neural mass model using resting fMRI connectivity and EEG microstates. *Front Comput Neurosci.* (2020) 13:91. 10.3389/fncom.2019.00091 32009922 PMC6978716

[36] Abreu R Jorge J Leal A Koenig T Figueiredo PEEG. Microstates predict concurrent fMRI dynamic functional connectivity states. *Brain Topogr.* (2021) 34:41–55. 10.1007/s10548-020-00805-1 33161518

[37] Philip RCM Dauvermann MR Whalley HC Baynham K Lawrie SM Stanfield AC. A systematic review and meta-analysis of the fMRI investigation of autism spectrum disorders. *Neurosci Biobehav Rev.* (2012) 36:901–42. 22101112 10.1016/j.neubiorev.2011.10.008

[38] Jia H Yu D. Aberrant intrinsic brain activity in patients with autism spectrum disorder: insights from EEG microstates. *Brain Topogr.* (2019) 32:295–303. 10.1007/s10548-018-0685-0 30382452

[39] D’Croz-Baron DF Baker M Michel CM Karp T. EEG microstates analysis in young adults with autism spectrum disorder during resting-state. *Front Hum Neurosci.* (2019) 13:173. 10.3389/fnhum.2019.00173 31244624 PMC6581708

[40] Jan RK Rihs TA Kojovic N Sperdin HF Franchini M Custo A Neural processing of dynamic animated social interactions in young children with autism spectrum disorder: a high-density electroencephalography study. *Front Psychiatry.* (2019) 10:582. 10.3389/fpsyt.2019.00582 31507462 PMC6714589

[41] Takarae Y Zanesco A Keehn B Chukoskie L Müller RA Townsend J. EEG microstates suggest atypical resting-state network activity in high-functioning children and adolescents with autism spectrum development. *Dev Sci.* (2022) 25:e13231. 10.1111/desc.13231 35005839

[42] Nagabhushan Kalburgi S Whitten AP Key AP Bodfish JW. Children with autism produce a unique pattern of EEG microstates during an eyes closed resting-state condition. *Front Hum Neurosci.* (2020) 14:288. 10.3389/fnhum.2020.00288 33132865 PMC7579608

[43] Bochet A Sperdin HF Rihs TA Kojovic N Franchini M Jan RK Early alterations of large-scale brain networks temporal dynamics in young children with autism. *Commun Biol.* (2021) 4:968. 10.1038/s42003-021-02494-3 PMC8367954 34400754

[44] Cheng W Rolls ET Gu H Zhang J Feng J. Autism: reduced connectivity between cortical areas involved in face expression, theory of mind, and the sense of self. *Brain.* (2015) 138:1382–93. 10.1093/brain/awv051 25795704 PMC4407191

[45] Leung MK Lau WKW. Resting-state abnormalities of posterior cingulate in autism spectrum disorder. *Prog Mol Biol Transl Sci.* (2020) 173: 139–59. 32711808 10.1016/bs.pmbts.2020.04.010

[46] Xu J Wang H Zhang L Xu Z Li T Zhou Z Both hypo-connectivity and hyper-connectivity of the insular subregions associated with severity in children with autism spectrum disorders. *Front Neurosci.* (2018) 12:234. 10.3389/fnins.2018.00234 29695950 PMC5904282

[47] Milz P Pascual-Marqui RD Achermann P Kochi K Faber PL. The EEG microstate topography is predominantly determined by intracortical sources in the alpha band. *NeuroImage.* (2017) 162:353–61. 10.1016/j.neuroimage.2017.08.058 28847493

[48] Pascual-Marqui RD Lehmann D Faber P Milz P Kochi K Yoshimura M The resting microstate networks (RMN): cortical distributions, dynamics, and frequency specific information flow. *arXiv* [preprint]. (2014). 10.48550/arXiv.1411.1949 35895330

[49] Dichter GS Felder JN Bodfish JW. Autism is characterized by dorsal anterior cingulate hyperactivation during social target detection. *Soc Cogn Affect Neurosci.* (2009) 4:215–26. 10.1093/scan/nsp017 19574440 PMC2728636

[50] Pereira AM Campos BM Coan AC Pegoraro LF de Rezende TJR Obeso I Differences in cortical structure and functional MRI connectivity in high functioning autism. *Front Neurol.* (2018). 9:539. 10.3389/fneur.2018.00539 30042724 PMC6048242

[51] Padmanabhan A Lynch CJ Schaer M Menon V. The default mode network in autism. *Biol Psychiatry Cogn Neurosci Neuroimaging.* (2017) 2:476–86. 29034353 10.1016/j.bpsc.2017.04.004 PMC5635856

[52] de Bock R Mackintosh AJ Maier F Borgwardt S Riecher-Rössler A Andreou C. EEG microstates as biomarker for psychosis in ultra-high-risk patients. *Transl Psychiatry.* (2020) 10:1–9. 10.1038/s41398-020-00963-7 32839449 PMC7445239

[53] Bagdasarov A Roberts K Bréchet L Brunet D Michel CM Gaffrey MS. Spatiotemporal dynamics of EEG microstates in four- to eight-year-old children: age- and sex-related effects. *Dev Cogn Neurosci.* (2022) 57:101134. 10.1016/j.dcn.2022.101134 35863172 PMC9301511

[54] Tomescu MI Rihs TA Rochas V Hardmeier M Britz J Allali G From swing to cane: sex differences of EEG resting-state temporal patterns during maturation and aging. *Dev Cogn Neurosci.* (2018) 31:58–66. 10.1016/j.dcn.2018.04.011 29742488 PMC6969216

[55] Loomes R Hull L Mandy WPL. What is the male-to-female ratio in autism spectrum disorder? A systematic review and meta-analysis. *J Am Acad Child Adolesc Psychiatry.* (2017) 56:466–74. 10.1016/j.jaac.2017.03.013 28545751

[56] Andreou C Faber PL Leicht G Schoettle D Polomac N Hanganu-Opatz IL Resting-state connectivity in the prodromal phase of schizophrenia: insights from EEG microstates. *Schizophr Res.* (2014) 152:513–20. 10.1016/j.schres.2013.12.008 24389056

[57] Strelets V Faber PL Golikova J Novototsky-Vlasov V Koenig T Gianotti LRR Chronic schizophrenics with positive symptomatology have shortened EEG microstate durations. *Clin Neurophysiol Off J Int Fed Clin Neurophysiol.* (2003) 114:2043–51. 10.1016/s1388-2457(03)00211-6 14580602

[58] Gschwind M Hardmeier M Van De Ville D Tomescu MI Penner IK Naegelin Y Fluctuations of spontaneous EEG topographies predict disease state in relapsing-remitting multiple sclerosis. *NeuroImage Clin.* (2016) 12:466–77. 10.1016/j.nicl.2016.08.008 27625987 PMC5011177

[59] Nunes AS Peatfield N Vakorin V Doesburg SM. Idiosyncratic organization of cortical networks in autism spectrum disorder. *NeuroImage.* (2019) 190: 182–90. 29355768 10.1016/j.neuroimage.2018.01.022

[60] Rajkumar R Régio Brambilla C Veselinović T Bierbrier J Wyss C Ramkiran S Excitatory–inhibitory balance within EEG microstates and resting-state fMRI networks: assessed via simultaneous trimodal PET–MR–EEG imaging. *Transl Psychiatry.* (2021) 11:60. 10.1038/s41398-020-01160-2 33462192 PMC7813876

[61] Rubenstein JLR Merzenich MM. Model of autism: increased ratio of excitation/inhibition in key neural systems. *Genes Brain Behav.* (2003) 2:255–67. 14606691 10.1034/j.1601-183x.2003.00037.x PMC6748642

[62] Malaia E Bates E Seitzman B Coppess K. Altered brain network dynamics in youths with autism spectrum disorder. *Exp Brain Res.* (2016) 234:3425–31. 10.1007/s00221-016-4737-y 27465558 PMC5097108

[63] Portnova GV Ivanova O Proskurnina EV. Effects of EEG examination and ABA-therapy on resting-state EEG in children with low-functioning autism. *AIMS Neurosci.* (2020) 7:153–67. 10.3934/Neuroscience.2020011 32607418 PMC7321768

[64] Brunet D Murray MM Michel CM. Spatiotemporal analysis of multichannel EEG: CARTOOL. *Comput Intell Neurosci.* (2011) 2011:e813870. 10.1155/2011/813870 21253358 PMC3022183

[65] Poulsen AT Pedroni A Langer N Hansen LK. Microstate EEGlab toolbox: an introductory guide. *biorxiv* [Preprint]. (2018). 10.1101/289850

